# American Association for Cancer Research Project Genomics Evidence Neoplasia Information Exchange: From Inception to First Data Release and Beyond—Lessons Learned and Member Institutions’ Perspectives

**DOI:** 10.1200/CCI.17.00083

**Published:** 2018-02-16

**Authors:** Christine M. Micheel, Shawn M. Sweeney, Michele L. LeNoue-Newton, Fabrice André, Philippe L. Bedard, Justin Guinney, Gerrit A. Meijer, Barrett J. Rollins, Charles L. Sawyers, Nikolaus Schultz, Kenna R. Mills Shaw, Victor E. Velculescu, Mia A. Levy, on behalf of the AACR Project GENIE Consortium

**Affiliations:** **Christine M. Micheel**, **Michele L. LeNoue-Newton**, and **Mia A. Levy**, Vanderbilt University Medical Center, Nashville, TN; **Shawn M. Sweeney**, American Association for Cancer Research, Philadelphia, PA; **Fabrice André**, Institut Gustave Roussy, Villejuif, France; **Philippe L. Bedard**, University of Toronto, Toronto, Ontario, Canada; **Justin Guinney**, Sage Bionetworks, Seattle, WA; **Gerrit A. Meijer**, Netherlands Cancer Institute, Amsterdam, the Netherlands; **Barrett J. Rollins**, Dana-Farber Cancer Institute, Brigham and Women’s Hospital, and Harvard Medical School, Boston, MA; **Charles L. Sawyers** and **Nikolaus Schultz**, Memorial Sloan Kettering Cancer Center, New York, NY; **Charles L. Sawyers**, Howard Hughes Medical Institute, Chevy Chase, MD; **Victor E. Velculescu**, Johns Hopkins University School of Medicine, Baltimore, MD; and **Kenna R. Mills Shaw**, The University of Texas MD Anderson Cancer Center, Houston, TX.

## Abstract

The American Association for Cancer Research (AACR) Project Genomics Evidence Neoplasia Information Exchange (GENIE) is an international data-sharing consortium focused on enabling advances in precision oncology through the gathering and sharing of tumor genetic sequencing data linked with clinical data. The project’s history, operational structure, lessons learned, and institutional perspectives on participation in the data-sharing consortium are reviewed. Individuals involved with the inception and execution of AACR Project GENIE from each member institution described their experiences and lessons learned. The consortium was conceived in January 2014 and publicly released its first data set in January 2017, which consisted of 18,804 samples from 18,324 patients contributed by the eight founding institutions. Commitment and contributions from many individuals at AACR and the member institutions were crucial to the consortium’s success. These individuals filled leadership, project management, informatics, data curation, contracts, ethics, and security roles. Many lessons were learned during the first 3 years of the consortium, including on how to gather, harmonize, and share data; how to make decisions and foster collaboration; and how to set the stage for continued participation and expansion of the consortium. We hope that the lessons shared here will assist new GENIE members as well as others who embark on the journey of forming a genomic data–sharing consortium.

## INTRODUCTION

The American Association for Cancer Research (AACR) Project Genomics Evidence Neoplasia Information Exchange (GENIE) is an international genomic data–sharing consortium focused on enabling advances in the understanding and treatment of cancer. The consortium’s first data release was on January 5, 2017, and an article that explored the scientific rationale for the project, the data standardization process, the landscape of the first publicly released data set, and future challenges was published.^1^ Now, the consortium has moved on to the work of preparing its next data releases, deciding which data fields to add, and reviewing applications for new members. With that work in mind, this review highlights some of the practical and administrative aspects of starting and running the GENIE consortium from the perspectives of AACR and the initial eight member institutions. We hope that the lessons shared here will assist new GENIE members and others who embark on the journey of forming a genomic data–sharing consortium.

## INCEPTION

**Fig 1.**
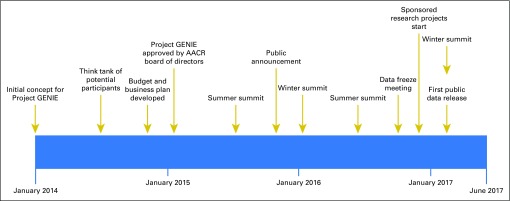
American Association for Cancer Research (AACR) Project Genomics Evidence Neoplasia Information Exchange (GENIE) timeline. AACR Project GENIE has developed rapidly from an initial concept in early 2014 to a formal business plan and approval by the AACR board of directors in January 2015. The consortium meets twice a year in January and July. Other notable dates are the November 2015 public launch, the October 2016 data freeze meeting, and the January 2017 first public release of data.

In 2014, Getz and colleagues of the Broad Institute estimated that to discover all potentially actionable mutations that occur at a prevalence of approximately ≥1%, approximately 5,000 samples per tumor type would need to be sequenced and analyzed.^2^ On the basis of this article and his experiences as AACR president and Global Alliance for Genomics and Health steering committee member and with the clinical sequencing program at Memorial Sloan Kettering Cancer Center, Charles L. Sawyers, MD, proposed the concept for AACR Project GENIE. He championed the premise that no single institution would be able to sequence a sufficient number of patients to improve clinical decision making in all tumor types, particularly in rare cancers and for rare variants in common cancers, and that the solution was to pool data from multiple institutions and share it publicly. In June 2014, AACR convened a think tank to gauge interest and begin to define the structure of what would become AACR Project GENIE (Fig 1). A key early decision was to focus on genomic data from high-quality clinical laboratories to increase reliability of mutation calls, not require reanalysis by a centralized institution, and result in a much greater number of patients with matching genomic clinical information than from research efforts. Between July and December 2014, a small team developed the business plan and budget for consideration by the AACR board of directors in January 2015. After board approval, the founding institutions operationalized the consortium by working through the technical aspects of data sharing and fund-raising. The fund-raising yielded three primary revenue sources: philanthropic gifts, commercial sponsorship of clinical studies, and grants.

## ADMINISTRATION

### Participating Institutions

AACR Project GENIE is executed through a coordinating center that comprised a director, clinical data manager, project manager, and program coordinator. The coordinating center staff is crucial to ensuring that the project is fully operational. Eight founding data-contributing institutions were chosen to provide a manageable number of institutions that could provide sufficient data to answer a clinical question within 2 years. Of note, these institutions had ongoing CLIA/ISO (Clinical Laboratory Improvement Amendments/International Organization for Standardization)–certified clinical sequencing programs in 2014 as well as expertise in the clinical and bioinformatics challenges of converting clinical data into knowledge. Furthermore, the founding institutions expressed a willingness to share clinical cancer genomic data and longitudinal outcomes and had institutional support to do so. A requirement of membership in the GENIE consortium is that each institution contributes at least 500 genomic records annually. The genomic data are accompanied by a minimum set of required clinical data elements. All institutions signed a master participation agreement and data use agreement before data submission. All institutions are required to protect and maintain patient privacy, which generally was done through existing consent for sharing deidentified data, specific consents for sharing specifically to GENIE, or a waiver of consent from the local institutional review board (IRB).^1^ In addition, all institutions are required to meet data standards and timelines and to participate in meetings and on committees. The founding institutions of AACR Project GENIE are the Dana-Farber Cancer Institute (Boston, MA); Institut Gustave Roussy (Paris-Villejuif, France); Netherlands Cancer Institute (Amsterdam, the Netherlands), on behalf of the Center for Personalized Cancer Treatment (Utrecht, the Netherlands); Johns Hopkins Sidney Kimmel Comprehensive Cancer Center (Baltimore, MD); Memorial Sloan Kettering Cancer Center (New York, NY); Princess Margaret Cancer Centre, University Health Network (Toronto, Ontario, Canada); The University of Texas MD Anderson Cancer Center (Houston, TX); and Vanderbilt-Ingram Cancer Center (Nashville, TN).

### Steering Committee

**Fig 2.**
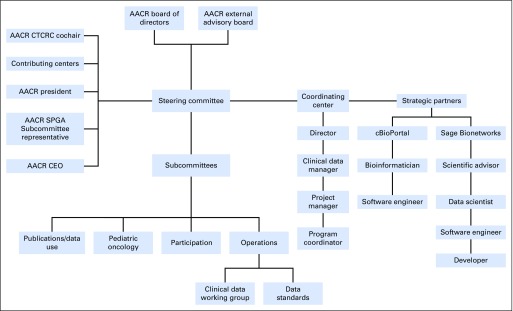
Organization of American Association for Cancer Research (AACR) Project Genomics Evidence Neoplasia Information Exchange (GENIE). The project is led by a steering committee that reports to the AACR board of directors and receives guidance from an external advisory board. Four subcommittees that make recommendations about various processes report to the steering committee, as does the AACR Project GENIE coordinating center. Project GENIE is executed in collaboration with two strategic partners: Sage Bionetworks and cBioPortal. CEO, chief executive officer; CTCRC, Clinical and Translational Cancer Research Committee; SPGA, Science Policy and Government Affairs.

AACR Project GENIE is led by a steering committee that comprises a senior representative from each contributing institution, the AACR chief executive officer, the current AACR president, one representative from the AACR Science Policy and Regulatory Affairs Subcommittee of the Science Policy and Government Affairs Committee, and one representative from the Clinical and Translational Cancer Research Committee (Fig 2). The steering committee reports to the AACR board of directors and receives oversight from an external advisory board of 11 individuals from industry, academia, and government with expertise in genomics and clinical data. The steering committee makes the final decision on all operational matters, with each European member receiving 2.5 votes to balance the geographic disparity in voting. All steering committee decisions can be appealed once. Decisions are attached as resolutions to the master participation agreement.

### Subcommittees

Three subcommittees report to the steering committee. The operations subcommittee prioritizes overall project efforts and ensures that all clinical projects and other project workflows run on schedule or that contingencies have been implemented. The participation subcommittee evaluates requests for new participating institutions and collaborations and ensures ongoing compliance of existing participants. The data use and publications subcommittee reviews concept proposals, manuscripts, and abstracts to minimize conflict with regard to research topics and authorship.

More subcommittees can be added; for example, a business development subcommittee is being created. In addition, a pediatric subcommittee has been partially established to provide guidance to the steering committee on pediatric oncology and to serve as a liaison between GENIE and the pediatric oncology community.

### Working Groups

In addition to the more formal subcommittees, working groups achieve specific tasks. A permanent clinical data working group identifies and defines the routinely collected clinical data elements. In addition, a data standards working group defines and ensures compliance with data formats,^1,3^ quality, quantity, and submission deadlines. Both groups report to the operations subcommittee. Examples of temporary working groups are the data analysis working group, which helped to clean and analyze the first data set for public release and for incorporation into the manuscript, and the manuscript working group, which wrote the manuscript and shepherded it through internal and external review.

### Sponsored Studies

One of the primary sources of project funding is sponsored studies. Participation in clinical studies is strongly encouraged when feasible but is not a requirement for consortium membership. Such studies are executed through ad hoc working groups that comprise study co-principal investigators (PIs), site PIs, site leaders, data abstractors, and statistical services. Statistical services usually reside within one of the co-PIs’ home institutions. We discovered that central overall project management of each sponsored study is essential to timely delivery of project milestones. Member institutions have faced a few unanticipated challenges, such as the need to understand and comply with confidentiality agreements between clinical trial sponsors and the individual institutions.

### Meetings

Communication has been essential to the GENIE consortium’s success, and meetings are a critical communication vehicle. The steering committee and other groups previously described meet regularly through Web conferencing. Twice a year, the steering committee and individuals from each participating institution meet at an in-person summit to discuss progress and plans. In addition to the operational and scientific value of these approximately 40-person meetings, the summits help to strengthen the consortium through shared activities and meals, and they encourage the generation of new ideas and formation of new collaborations. In addition to the summits, ad hoc in-person meetings occasionally are required. One example was the data “hackathon” where a group assembled to finalize the data and begin the manuscript. The early meetings focused on building the infrastructure and operational procedures necessary for GENIE to function. Now that the project is more mature, we recently engaged in a facilitated strategic planning session in July 2017 to chart the project’s future. One of the lessons learned is that there is never enough time to adequately discuss scientific questions at in-person summits, and participation is limited to a small number of attendees from each institution. To address this issue, we may institute a scientific symposium.

### Expansion of the Consortium

After the first public data release, an open call for new sites was broadly publicized, including direct communication with each institution that had inquired about joining. The application process was handled through an online survey that opened March 8, 2017, and closed May 1, 2017. Any institution that began an application before the close date or requested an extension was granted an extension to May 15, 2017. The survey results were reviewed by the participation subcommittee by using criteria published on the GENIE Web site.^4^ Any unresolved issues or follow-up questions were handled on an applicant-by-applicant basis. Finalists were then given instructions to complete a sample data upload to identify potential issues in interacting with the project data platforms. The criteria for new sites are the same as for the founding institutions and are described in Participating Institutions and listed in Appendix Table A1. Future rounds of expansion are anticipated to add more qualified institutions to the GENIE consortium.

## DATA MANAGEMENT

**Fig 3.**
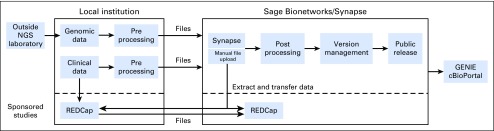
Technology and architecture, which shows how data flow in the consortium, including the location of data and the technologies used. GENIE, Genomics Evidence Neoplasia Information Exchange; NGS, next-generation sequencing; REDCap, Research Electronic Data Capture.

Several open-source technologies allow GENIE to share and analyze data safely and securely both internally and externally (Fig 3). The Synapse platform from Sage Bionetworks (Seattle, WA; www.synapse.org) provides data versioning and provenance and serves as the primary data hub for the consortium.^5^ For sponsored studies, clinical data are collected and staged by using the open-source Research Electronic Data Capture (REDCap) system^6^ (www.project-redcap.org); these data are subsequently imported into Synapse. All data are pushed into cBioPortal^7,8^ (www.cbioportal.org/genie) for data visualization and analysis; data also can be directly exported from both Synapse and REDCap for analysis in other platforms. In addition, each technology permits multiple levels of access control so that public, consortium, and sponsor access to the appropriate data sets is easily managed on all platforms; data access is centrally managed by Sage Bionetworks.

### Genomic Data

All genomic data are mapped to ref 37 (hg19). Note that the Genomic Data Commons (GDC) uses GRCh38, and we likely need to begin to transition GENIE in this direction. The consortium only shares variant calls and not raw data, which was one of the early agreements that facilitated data sharing. For each gene panel, a browser extensible data (BED) file is provided, which is a list of all the genomic features that a particular test sequences. Each site defines a workflow file that links information about the sequencing assay to the BED file. The BED and workflow files may not be straightforward to obtain if an institution uses outside vendors for its sequencing and if these file types are not included in the data use agreements between institutions and vendors.

After the initial data were available to the consortium, an ad hoc working group analyzed the initial data freeze for the manuscript and first public release. As part of this exercise, a few initial issues were identified, including mutations in *cis* (which occurs when two variants in proximity are reported with similar allele fraction), adherence to variant call format, normalization of gene symbols, and the need to develop a germline filter for tumor-only sequencing.^1^ The filter removes as many private or noncommon single-nucleotide polymorphisms as possible. Although the filter is effective, we could not be sure that all risk of patient reidentification was removed; thus, we implemented terms of use that must be reviewed and endorsed before users can access data.

Currently, only CLIA/ISO-certified data are eligible for submission to GENIE. The guidelines for sequencing varied somewhat between institutions,^3^ and this is summarized in Table 1. Submitted data are rejected if they fail validation with tools developed collaboratively between Sage Bionetworks and scientists at participating institutions. In addition, although the central GENIE database hosted by Sage Bionetworks contains all mutation data reported by each center, cBioPortal currently excludes silent, intronic, 3′ untranslated region, 3′ flank, 5′ untranslated region, 5′ flank, and intergenic region mutations; however, these will be made available in cBioPortal in the near future.

**Table 1.**
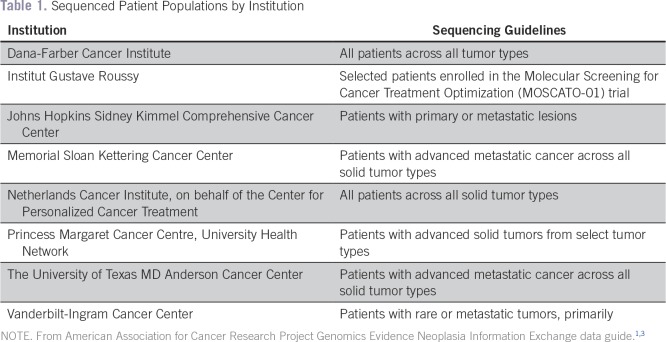
Sequenced Patient Populations by Institution

### Clinical Data

The consortium took a pragmatic approach to getting started and began with a limited set of clinical data elements that was informative but easily extracted in a short period.^3^ Now that the consortium is well established, it is conducting feasibility analyses to add more clinical data elements. Our most important near-term additions are slated to be age at death, age at last follow-up, and institutionally administered cancer-directed therapeutics, with others to follow.

A great variability exists among the institutions with respect to the ability to pull data automatically versus through manual chart review for the minimum clinical data elements. Among those institutions that required chart review, considerable differences existed with respect to dedicated resources for curation. Even among institutions with more automated extraction and thorough clinical annotation of all institutional patients, there was a general underestimation of the time required to deliver data because of field mapping issues, retrieval of missing data, recoding of pathologic diagnosis to the project ontology, and so forth.

### Data for Sponsored Studies

Sponsored studies generally require manual extraction of additional data elements from the electronic health record of the local institutions. One half of the consortium is able to share limited protected health information (PHI)—dates, specifically—for purposes of sponsored studies, whereas the remaining one half cannot. As a result, copies of study-specific data dictionaries are implemented in local instances of REDCap hosted behind the firewalls of institutions that cannot share limited PHI. After data collection, the data are deidentified for export to Synapse. The remaining sites collect data in a centralized REDCap instance held by Sage Bionetworks. PHI is deidentified before delivery to study sponsors. As the project continues to mature, the consortium aims to automate extraction of more clinical data elements.

### Data Redistribution

The consortium is deeply committed to open science and data sharing as long as patient privacy is protected. To this end, terms of access have been implemented that require steering committee approval of any data redistribution, which is predicated on the ability of the receiving platform to implement the same terms of access that GENIE has implemented. To date, five requests for redistribution have been received and granted. As good global citizens, the consortium has deposited its first data release, version 1.0.1, in the National Cancer Institute GDC.^9^ Our interactions with GDC were straightforward, and because of the submission it has already received from Foundation Medicine, GDC is prepared to accept a subset of clinical data and to help with mapping. The most difficult aspect of the submission was the database of genotype phenotype registration.

### Data Access and Sharing

Several tiers of data access are built into the master participation agreement. After GENIE is fully operationalized, institutions will have sole access to their own data for 6 months from the date of the sequencing report. Then, on a twice-yearly basis, genomic and clinical data will be deposited for all samples sequenced 6 months ago. Consortium members will have access to the data for 6 months, after which the data will be released to the public. For the first public data release, GENIE members waived their consortium-only access.

**Fig 4.**
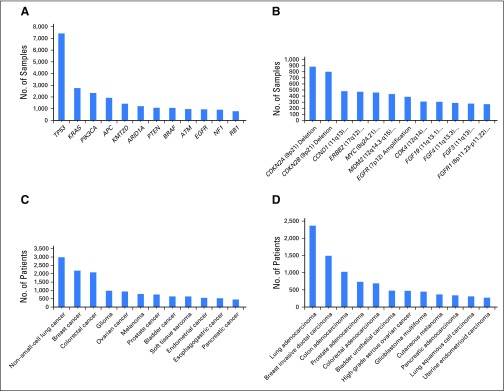
Overview of the Genomics Evidence Neoplasia Information Exchange (GENIE) version 1.0.1 data set. (A) The top 12 mutated genes, (B) copy number alterations, (C) major cancer types, and (D) cancer subtypes from the first public data release are plotted.

The initial public release of GENIE data occurred on January 5, 2017, and consisted of 18,804 samples from 18,324 patients contributed by the eight founding institutions. The data came from 12 unique next-generation sequencing panels that ranged from 46 to 429 genes and covered 1,040 unique genes. Of note, the most commonly mutated genes in the population are *TP53*, *KRAS*, and *PIK3CA*, respectively, with *CDKN2A* and *CDKN2B* deletions and *CCND1* amplification representing the most common copy number alterations. The three most common cancers are non–small-cell lung cancer, breast cancer, and colorectal cancer, respectively, with lung adenocarcinoma, invasive ductal carcinoma, and colon adenocarcinoma comprising the most common cancer subtypes (Fig 4).

The public can access the data either through the GENIE-specific instance of cBioPortal or download the individual data sets for manipulation as the user sees fit directly from Sage Bionetworks.^10^ As of September 5, 2017, 2,608 individuals had requested access through cBioPortal, and up to 557 individuals had downloaded one of the files from Synapse (Appendix Table A2). In addition, data from Google Scholar (Google, Mountain View, CA) show that two articles and a thesis have referenced the landscape article.

## INSTITUTIONAL PERSPECTIVES

Each institution that joined GENIE underwent an internal cost-benefit analysis, legal and administrative tasks related to joining the consortium, and work to pull and process data submitted to GENIE. This section describes the institutional perspectives of participation in the GENIE consortium.

### Value to the Institution

Institutions joined the consortium because of the value of pooling local genomic and clinical data to generate larger data sets with sufficient power to study rare variants or rare diseases. In addition, many institutions view participation in data-sharing initiatives as reinforcing of organizational reputations as leading cancer centers and as an extension of their missions. Institutions also participate to be leaders in the field, solve the myriad challenges associated with data sharing, and help future participants in this and other data-sharing initiatives by developing and sharing best practices. Finally, many institutions have found participation to be an excellent opportunity to organize their own data and processes as well as to collaborate with other institutions.

Institutions remain in the consortium because they value the cross-institutional pollination and commitment to public data sharing as members of the “community of the willing.” Participation in GENIE has been a stimulating and instructive experience where lessons learned have been immediately applied to improve internal processes. Furthermore, researchers at many institutions anticipate opportunities to conduct research on the basis of GENIE data and look forward to taking advantage of the institution- and consortium-only access built into future submissions and releases of data sets.

### Institutional Stakeholders

Each institution noted the many individuals involved in the project who played a range of roles, including leadership, project management, informatics, contracts, IRB membership, security, pathology, clinical services, clinical genetics, and data curation. All underscore the requirement for commitment from institutional leadership to participate; however, buy-in from the clinicians and researchers who generate and want to use the data is essential. There is a need to engage this group to address concerns before sharing data. The institutions have taken different approaches, but those who have used an internal point person (or site lead) as the primary point of contact between the GENIE coordinating center and other internal stakeholders reported this as an effective way of managing the consortium relationship.

One of the primary lessons shared by the participating institutions was the need for an institutional champion. Being a part of the consortium requires a commitment of financial and institutional resources, including personnel, and executive leadership must be supportive of the endeavor if it is to succeed. Each institution ultimately must be committed to sharing broad clinical data beyond the basic elements. Furthermore, a well-organized institutional team is required to ensure that milestones are met.

### Institutional Environmental Factors

When thinking about participation in a data-sharing consortium, institutions should consider the ability of their electronic health record to structure data so that automated data extraction is possible. Institutions can take stock of their processes and map these against the core processes of the data-sharing consortium and address any gaps. The dedication of resources to create systems to automate or structure essential data elements early will save time and resources later and make sharing data multiple times a year easier. Even so, each institution needs to dedicate resources to do the technical work of data manipulation for integration into the target. Many institutions were surprised by the resource requirements and initially under-resourced the project.

Institutions receive partial compensation from AACR to cover expenses incurred as a result of their participation in the consortium and use a combination of existing operational funds, small grants, and philanthropy to cover remaining costs. The Netherlands Cancer Institute and MD Anderson Cancer Center have additional funding from the Dutch Ministry of Health and Dutch Cancer Society and the Khalifa Institute for Personalized Cancer Therapy, respectively. An in-depth accounting of the actual costs of membership has yet to be undertaken; however, direct costs of sequencing range from several to tens of millions of US dollars in addition to significant donations of uncompensated personnel time.

With respect to ethical, legal, and social implications of the project, exchange of best practices with regard to IRB waivers and informed consent documents may aid members and potential members to get their own ethical, legal, and social implication issues resolved in advance. Numerous ethical, legal, and logistic challenges are associated with international data transfer. One of these concerned the invalidation by the European Court of Justice of the safe harbor procedure for exchange of personal data in 2015, which means that alternative procedures had to be implemented to allow the European partners to continue their participation in GENIE. Furthermore, the consortium encountered intellectual property issues related to clinical trial–associated data, such as disclosure of treatment with investigational agents. Discussion and consideration of these hurdles early and a consensus on data elements to be collected and how to handle potential intellectual property and PHI concerns are important. Overall, institutions were pleasantly surprised at how willing all were to work together and share data.

## DISCUSSION

At the time of the inception of AACR Project GENIE in 2014, few mature cancer data consortia existed. Since, several have emerged with variance in focus and approach. AACR Project GENIE is distinguished from these in several notable ways. First, AACR Project GENIE is focused on cancers where tumors have already undergone a minimum depth of somatic sequencing, whereas several other consortia include all patients with cancer from an institution (eg, ASCO CancerLinQ, National Comprehensive Cancer Network/Flatiron, TriNetX). This focus has several advantages. At its inception, this more limited population for data sharing lowered the barrier to entry for many institutions who were not comfortable about sharing all their cancer data, and it significantly lowered the operational costs of the GENIE project because tumor sequencing as part of clinical care had already occurred as a condition of patient eligibility. Other consortia provide sequencing services as part of their program, some as commercial sequencing vendors (eg, Foundation Medicine’s Precision Medicine Exchange Consortium, Tempus, Caris Life Sciences) and others as part of the benefits of membership (eg, ORIEN [Oncology Research Information Exchange Network]). Although these consortia incur the additional costs of sequencing, they have the advantage of having a single sequencing platform over which to evaluate their population. Because the members of the GENIE consortium use various sequencing platforms, data normalization is required, and analyses must take into account variance in breadth of sequencing across patients. However, because sequencing data are structured and there was high concordance of data definitions and standards, data normalization was achieved relatively rapidly and at low cost. This focus resulted in the rapid generation of a highly valuable population of molecularly characterized cancer cases for discovery, even with the current limited depth of clinical annotation.

AACR Project GENIE has a strong commitment to public release of consortium data, which distinguishes it from all other non–federally funded cancer data consortia, and is reinforced by the Beau Biden Cancer Moonshot.^11,12^ Consortium members believe that the data will have a far greater impact on the cancer community if made publically available. Furthermore, AACR believes strongly that all AACR members should have access to these data, which is achieved through public release. However, public release of data requires additional safeguards for patient privacy, especially when genetics data are included. Given that most of the GENIE members’ next-generation sequencing panels tested tumor tissue only and not tumor-normal pairs, special processing was required to remove potential germline alterations in somatic tumor calls to decrease the risk of reidentification in the public data set. We hope that GENIE’s approach to public release of data will serve as a model to other consortia.

In conclusion, the AACR Project GENIE member institutions are excited to share their first public data release and look forward to continued participation in the consortium. Several lessons were learned related to creating and sustaining a data-sharing consortium, which are described here; Appendix Table A3 lists all the obstacles encountered. First, the move from project initiation to first data release took longer than anticipated, and the ability for the steering committee to deviate from the original contract and adapt to changing needs was important for the success of the project. Second, the sharing of genomic data was fairly easy and straightforward because of a high concordance of data definitions and standards and a structured electronic form from their generation, unlike much clinical data. The first data aggregation effort helped to uncover nuances that were subsequently resolved at the respective institutions, which we believe was a major benefit to these institutions and will facilitate ongoing data sharing. Third, a need exists for continual communication with institutional stakeholders. Careful records of decisions are needed, and communication of decisions should be broad and repeated. Fourth, the consortium currently is working through the challenges of setting up processes and resources for submitting data multiple times a year. Many lessons were learned during the first data submission, but institutions face additional challenges when planning and executing periodic data submissions. Finally, the building of trust among the institutions and with AACR is key. Even among the community of the willing, consensus or agreement is not a foregone conclusion; negotiation is necessary; and, as a result, solid relationships built on trust are essential.

## Figures and Tables

**Table A1.**
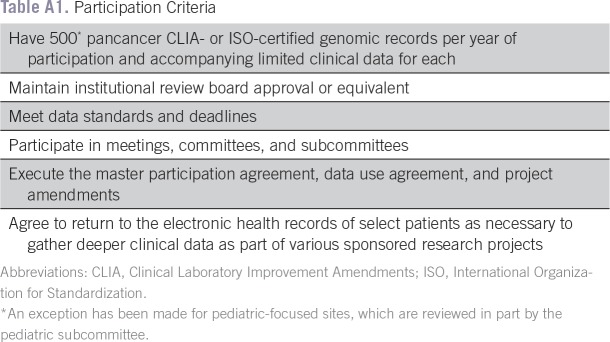
Participation Criteria

**Table A2.**
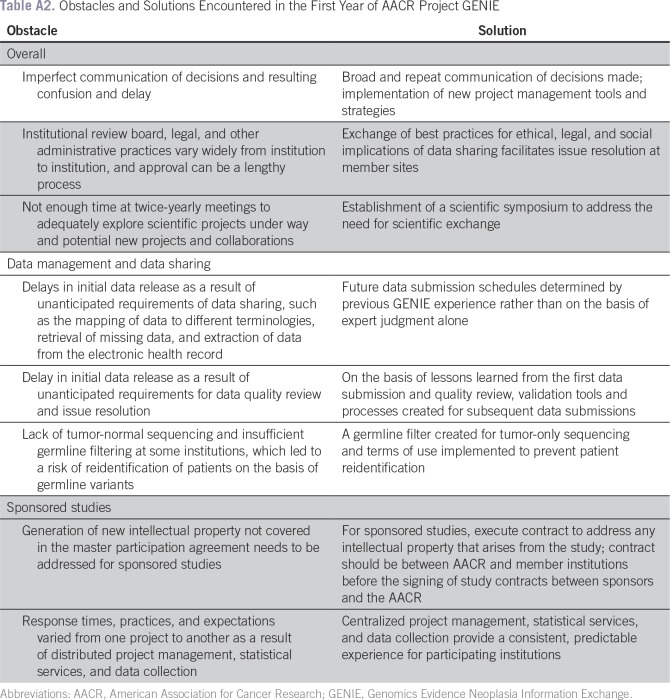
Obstacles and Solutions Encountered in the First Year of AACR Project GENIE

**Table A3.**
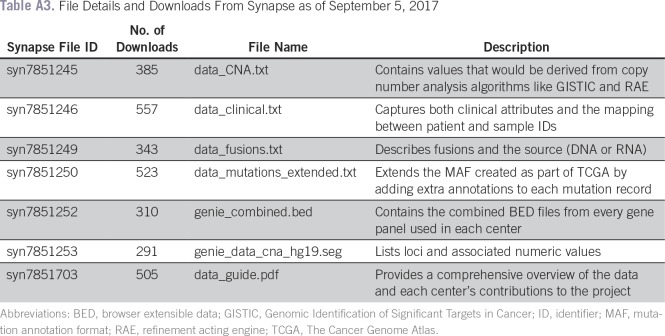
File Details and Downloads From Synapse as of September 5, 2017
